# Context‐Stratified Mendelian Randomization: Exploiting Regional Exposure Variation to Explore Causal Effect Heterogeneity and Nonlinearity

**DOI:** 10.1002/sim.70727

**Published:** 2026-09-01

**Authors:** Stephen Burgess, Benjamin A. R. Woolf, Amy M. Mason

**Affiliations:** ^1^ Medical Research Council Biostatistics Unit University of Cambridge Cambridge UK; ^2^ British Heart Foundation Cardiovascular Epidemiology Unit, Department of Public Health and Primary Care University of Cambridge Cambridge UK; ^3^ Medical Research Council Integrative Epidemiology Unit University of Bristol Bristol UK; ^4^ School of Psychological Science University of Bristol Bristol UK; ^5^ Victor Phillip Dahdaleh Heart and Lung Research Institute University of Cambridge Cambridge UK

**Keywords:** causal inference, effect heterogeneity, instrumental variables, meta‐regression, nonlinearity, stratification

## Abstract

Mendelian randomization (MR) uses genetic variants as instrumental variables (IVs) to make causal claims. Standard MR approaches typically report a single population‐averaged estimate, limiting their ability to explore effect heterogeneity or nonlinear dose–response relationships. Existing stratification methods, such as residual‐based and doubly‐ranked stratified MR, attempt to overcome this but rely on strong and unverifiable assumptions. We propose an alternative, context‐stratified Mendelian randomization, which exploits exogenous variation in the exposure across subgroups—such as recruitment centers, geographic regions, or time periods—to investigate effect heterogeneity and nonlinearity. Separate MR analyses are performed within each context, and heterogeneity in the resulting estimates is assessed using Cochran's Q statistic and meta‐regression. We demonstrate through simulations that the approach detects heterogeneity when present while maintaining nominal false positive rates under homogeneity when appropriate methods are used. In an applied example using UK Biobank data, we assess the effect of vitamin D levels on coronary artery disease risk across 20 recruitment centers. Despite some regional variation in vitamin D distributions, there is no evidence for a causal effect or heterogeneity in estimates. Compared to stratification methods requiring model‐based assumptions, the context‐stratified approach is simple to implement and unaffected by collider bias, provided the context variable is exogenous. However, the method's power and interpretability depend critically on meaningful exogenous variation in exposure distributions between contexts. In the example of vitamin D, subgroups from other stratification methods explored a much wider range of the exposure distribution.

## 1 Introduction

Mendelian randomization (MR) uses genetic variants as instrumental variables (IVs) to estimate the causal effect of a modifiable exposure on an outcome [1, 2]. Under the core IV assumptions (relevance, independence, and exclusion restriction), MR can provide unbiased causal estimates by leveraging randomness in the inheritance of genetic variants at conception as a natural experiment [3]. Conventional MR typically reports an average causal effect estimate for the population [4]. This is analogous to the average treatment effect in a randomized controlled trial, which is averaged over all individuals whose exposure value is altered by randomization [5].

However, causal effects may not be constant across all individuals or settings. In many cases, one may expect effect heterogeneity: the exposure might have a stronger effect in some subgroups of the population than others [6, 7]. Alternatively, a nonlinear dose–response relationship might exist [8]. In a randomized controlled trial, investigators often explore this by performing stratified analyses (also called subgroup analyses) to see if the treatment effect differs by baseline risk factors or other characteristics [9]. Identifying such heterogeneity is critical for targeting interventions to those who would benefit most. For example, if an exposure is only causally beneficial when baseline levels are low (a threshold effect), a population‐averaged estimate would fail to identify this important nuance.

Performing subgroup analyses in MR is challenging. Any covariate measured after conception is technically “post‐randomization” (since genetic randomization occurs at conception). Naively stratifying a MR analysis on a post‐randomization covariate (especially one affected by the genotype or exposure) can induce collider bias [10], potentially yielding biased stratum‐specific estimates [11]. For instance, stratifying on the exposure level itself (or a variable influenced by it) will induce correlations between the instrument and confounders within strata, invalidating estimates [12]. Exceptions to this rule are covariates such as age, sex, and ancestry, which are either determined by external factors or fixed from conception.

Recent methodological work has highlighted these concerns and proposed solutions for safer stratification in Mendelian randomization. In particular, the residual‐based and doubly‐ranked stratification methods have been developed to form exposure strata while preserving instrument validity [13, 14]. Under specific assumptions, these techniques ensure that genetic instruments remain valid instruments within each stratum, avoiding bias when exploring nonlinear effects. However, the assumptions that these methods make go beyond the standard IV assumptions, and violations of these assumptions can lead to bias and potentially to misleading findings [15, 16].

An alternative proposal is to investigate how the causal effect varies across subgroups that differ in their distribution of the exposure, but are defined in a way that is not based on the genetic variants or the exposure. A natural way to do this is to use structure within the data, such as recruitment centers, which we refer to as contexts. The central idea is simple: if the average level of the exposure differs between subgroups, then performing MR within each subgroup provides a causal estimate relevant to that exposure distribution. Comparing these estimates can reveal effect heterogeneity or nonlinearity. We refer to this approach as “context‐stratified Mendelian randomization.”

This approach treats context as analogous to a baseline effect modifier. It is conceptually akin to a multisite trial analyzing treatment effects separately at each site to see if they differ by some site‐level condition [17]. It is similarly analogous to meta‐regression, in which study‐specific estimates are regressed on a study covariate to investigate trends in estimates [18]. We note that while estimates in a randomized controlled trial (both in main analyses and in subgroup analyses stratified by baseline covariates) are typically unconfounded due to randomization, both comparisons between trial estimates and comparisons of subgroup estimates are typically confounded.

In this article, we first describe the methodology of context‐stratified MR (Section 2). We demonstrate through simulations that the approach can successfully identify nonlinear causal patterns and does not detect false heterogeneity when the true effect is homogeneous and appropriate methods are used (Section 3). We present an example of its application in UK Biobank, evaluating estimates of the effect of vitamin D levels on coronary artery disease risk in different recruitment centers, and assessing heterogeneity and trends in estimates across centers (Section 4). We then discuss the insights gained, the differences from other approaches, and the potential implications for epidemiological research and targeted public health interventions (Section 5). In particular, we discuss the interpretation of differences in estimates between contexts.

## 2 Methods

### 2.1 Concept

Context‐stratified MR involves splitting the study population into subgroups based on an external context variable that influences the exposure distribution. Typical context variables could be study regions, recruitment centers, time periods, age groups, or other pre‐specified subpopulations. The key assumptions are that these subgroups have different average levels of the exposure of interest, and that the context variable is exogenous; that is, it is not a function of any variable in the model. We then perform separate MR analyses within each context to obtain context‐specific causal estimates. By examining how these estimates differ across contexts, we can infer whether the causal effect might depend on the exposure distribution (suggesting effect modification or nonlinearity). This approach is not a robust method for MR, but an extension to consider a somewhat different causal question; although, as with standard MR, it relies on the validity of the IV assumptions.

### 2.2 Assumptions

Within each subgroup defined by the context variable, the standard IV assumptions must hold for any genetic variant used as an instrument:

1. **Relevance:** The genetic variant is associated with the exposure in each subgroup.
2. **Independence:** There is no confounding pathway between the variant and outcome in any subgroup.
3. **Exclusion restriction:** The variant can only affect the outcome through the exposure.

Because we stratify on a context variable that is external to the variables in the model, this stratification should not introduce collider bias. As analyses are performed separately in each subgroup, it is not necessary for the genetic variants to be distributed identically in the subgroups. However, if we want to attribute differences in context‐specific estimates to the exposure, then other factors should be comparable across subgroups.

### 2.3 Analytical Steps

The implementation of context‐stratified MR can be summarized as follows:

- Step 1: Select a context variable that partitions the dataset into K subgroups with distinct exposure distributions.
- Step 2: For each subgroup k, calculate a summarized measure of the exposure (for example, the mean or median exposure level). These will serve as indicators of the exposure distribution for interpretation and for meta‐regression modelling.
- Step 3: Within each subgroup k, conduct an IV analysis to estimate the causal effect of the exposure on the outcome. With a continuous outcome, this could be done via two‐stage least squares if individual‐level data are available or via the ratio method if a single genetic instrument is used. Standard summarized‐data methods such as the inverse‐variance weighted method could be used if context‐specific summarized data are available [19].
- Step 4: Compare the context‐specific IV estimates ${\hat{\beta }}_{1},{\hat{\beta }}_{2},\ldots ,{\hat{\beta }}_{K}$. A formal heterogeneity test can be performed using Cochran's Q statistic [20]. A significant Q statistic would indicate that the estimates are more variable than expected by chance, consistent with effect heterogeneity or nonlinearity.
- Step 5: To quantify and visualize the relationship between causal estimates and the exposure distribution, perform a meta‐regression. In a meta‐regression, the dependent variable is the vector of context‐specific IV estimates, and the key independent variable is the vector of average exposure levels in each subgroup. A positive slope, for instance, would indicate contexts with larger mean exposure levels have greater causal estimates (or vice versa). The trend can also be explored visually by a scatter plot of the context‐specific IV estimates against the mean exposure values.

### 2.4 Role of Generative Artificial Intelligence

Generative artificial intelligence (AI) was used to write the initial draft of this manuscript. The initial draft of this article was generated by the Deep Research feature of ChatGPT version 4. It was then substantially edited by human authors. We did ask ChatGPT to help with further editing; however, it tried to re‐write the manuscript in a way that was not constructive. Outputs from further prompts were not used in the drafting of the work. The initial prompt provided a suggested simulation study; however, this was inadequate and substandard, and so the simulation study used in the article is not based on this. The initial ChatGPT prompt is provided in the Supporting Information, as is the initial draft manuscript prepared by the model.

## 3 Simulation Study

### 3.1 Set‐Up

To benchmark the context‐stratified MR approach, we conducted a simulation study. We simulate a continuous exposure X, a genetic instrument G (taking values 0,1,2 to mimic an allele count), an unmeasured confounder U, and a continuous outcome Y. We generate data on 10 000 individuals in each of 10 contexts, making a total of 100 000 individuals. The mean level of the exposure is varied between contexts by adding a constant $\alpha_{k}$. We consider two scenarios for between‐context differences: a larger difference scenario where $\alpha_{k}$ is 8 in the first context, 8.2 in the second context, then 8.4 and so on up to 9.8, and a smaller difference scenario where $\alpha_{k}$ is 9 in the first context, 9.1 in the second context, then 9.2 and so on up to 9.9. We consider three scenarios for the true causal effect of the exposure on the outcome:

1. linear homogeneous effect (no effect modification or nonlinearity): f(x)=0.8x.
2. quadratic effect: $f(x)=0.04x^{2}$.
3. threshold effect: f(x)=0.25I(x>10)(x−10)

where I(x>10) is an indicator function that is 1 if the exposure is greater than 10, and 0 otherwise.

The initial data‐generating model for individual i in context k is as follows:

(1) $$\begin{array}{ll} g_{ik} & ∼\text{Binomial}(2,0.3)\\ u_{ik},\epsilon_{Xik},\epsilon_{Yik} & ∼𝒩(0,1)\;\text{independently}\\ x_{ik} & =\alpha_{k}+0.5g_{ik}+u_{ik}+\epsilon_{Xik}\\ y_{ik} & =f(x_{ik})-u_{ik}+\epsilon_{Yik} \end{array}$$

We generate 1000 simulated datasets for each of the six scenarios. For each simulated dataset, we calculate the kth context‐specific IV estimate using the ratio method as the genetic association with the outcome in context k divided by the genetic association with the exposure in context k. We then calculate Cochran's Q statistic as a measure of heterogeneity in the context‐specific IV estimates, and perform meta‐regression of the context‐specific IV estimates on the mean level of the exposure in each context.

Various versions of Cochran's Q statistic have been proposed, as the method relies on the standard errors of the estimates to use as weights, and there are different ways of calculating these standard errors. Cochran's Q statistic assumes that the estimates are normally distributed, but this assumption may be strained for IV estimates, as these (with a single instrument) are calculated as a ratio of coefficients, and the ratio of two normal random variables has no finite mean. We calculate Cochran's Q statistic and its associated p‐value in three ways: using first‐order weights, using modified second‐order weights [21], and using a permutation‐based approach as implemented in MR‐PRESSO package [22]. Code to run the simulation study is provided in the Supporting Information.

We perform the simulation study in three parts. First, we consider the null effect scenario (linear homogeneous effect) only, and investigate false positive rates (that is, Type 1 error rates) in this case. Second, we consider all three effect scenarios, and calculate results from heterogeneity and trend tests applied to context‐specific estimates. We compare these results to results for the same methods applied to stratum‐specific estimates from the residual‐based and doubly‐ranked methods. Third, we repeat the comparison of context‐specific and stratum‐specific estimates, except that we generate the exposure as:

(2) $$x_{ik}=\alpha_{k}+0.5g_{ik}+0.1g_{ik}\times u_{ik}+u_{ik}+\epsilon_{Xik}$$

so that the parametric assumptions in the residual‐based method (the constant‐effect assumption) and the doubly‐ranked method (the rank‐preserving assumption) are violated.

For each scenario, we report the proportion of simulated datasets in which heterogeneity was detected (defined as the p‐value for the Q statistic being less than 0.05), and the proportion of simulated datasets in which a trend was detected (defined as the p‐value for the mean exposure term in the meta‐regression being less than 0.05). The genetic instrument explained around 4.3% of the variance in the exposure (larger differences) or 4.8% of the variance (smaller differences), corresponding to an average F statistic of around 4500.

### 3.2 Results

**Coverage under the null:** Results from the simulation study in the null effect scenario are shown in Table 1. With larger differences, the proportion of simulated datasets in which heterogeneity is detected is 12.3% when using first‐order weights. This is greater than the expected 5% false positive rate that we would expect under the null hypothesis. A similar finding has been observed previously, in which the heterogeneity test over‐rejects the null even with conventionally strong instruments [21, 22]. When the heterogeneity test is being used to detect pleiotropy, over‐rejection of the test is tolerable, as between‐variant differences in estimates are interpreted as evidence suggesting pleiotropy. However, in this case, we do not want to over‐reject the null hypothesis. When using modified second‐order weights or the MR‐PRESSO permutation‐based approach, the proportion of simulated datasets in which heterogeneity is detected is 0.5%, well below the nominal 5% level. The proportion of datasets in which a trend is detected when the true effect is linear is 5.4%, close to the expected 5% level. Findings were similar with smaller differences, with elevated coverage rates for the heterogeneity test using first‐order weights of 11.5%, conservative rates for the heterogeneity test using modified second‐order weights of 0.5% and MR‐PRESSO of 0.1%, and close to nominal levels for the trend test of 6.0%.

**TABLE 1:** Simulation study results: proportion of simulated datasets in which either heterogeneity or a trend was detected (defined as the p‐value being less than 0.05) with the null model for the effect of the exposure on the outcome (that is, linear homogeneous effect) and two sets of values of the differences between groups.

Null effect scenarios	Larger differences	Smaller differences
(Linear homogeneous effect)	between subgroups	between subgroups
Cochran's Q test, first‐order weights	12.3%	11.5%
Cochran's Q test, modified second‐order tests	0.5%	0.5%
Cochran's Q test, MR‐PRESSO implementation	0.5%	0.1%
Trend test	5.4%	6.0%

**Comparison of approaches:** Results in null and positive effect scenarios are shown in Table 2. Results for the context‐stratified method differ slightly from those in Table 1 due to Monte Carlo error arising from the limited number of simulations. However, the same findings are observed throughout, with heterogeneity tests either under‐rejecting (for first‐order weights) or over‐rejecting (for modified second‐order weights and the MR‐PRESSO implementation) under the null. In contrast, in the quadratic and threshold scenarios, the proportion of simulated datasets correctly rejecting the null is well over 5% for both versions of the heterogeneity test, and for the trend test. We do not interpret these numbers too strongly, as power to detect differences in estimates depends on many factors, including sample size, the difference in the exposure distribution between subgroups, and the instrument strength. However, we note that this is a fairly optimistic setting, in which the instrument is strong, the sample size is large, and differences in the exposure distribution between subgroups are substantial. Even in this setting, power to detect differences between estimates is far from perfect.

**TABLE 2:** Simulation study results: proportion of simulated datasets in which either heterogeneity or a trend was detected (defined as the p‐value being less than 0.05) with three different models for the effect of the exposure on the outcome (i.e., linear homogeneous effect) and two sets of values of the differences between groups, comparing results from context‐based stratification to previous methods for nonlinear Mendelian randomization.

	Larger differences			Smaller differences		
	between subgroups			between subgroups		
	Linear	Quadratic	Threshold	Linear	Quadratic	Threshold
Context‐based stratification						
First‐order	13.0%	60.1%	39.8%	11.1%	28.2%	19.1%
Modified second‐order	0.7%	32.2%	39.1%	0.2%	1.6%	18.5%
MR‐PRESSO test	0.4%	30.3%	38.9%	0.2%	1.5%	17.0%
Trend test	5.2%	60.5%	59.6%	4.6%	34.2%	26.1%
Residual‐based stratification						
First‐order	5.4%	100.0%	100.0%	5.5%	100.0%	100.0%
Modified second‐order	4.7%	100.0%	100.0%	4.9%	100.0%	100.0%
MR‐PRESSO test	3.9%	100.0%	100.0%	4.3%	100.0%	100.0%
Trend test	5.0%	100.0%	99.9%	4.4%	100.0%	100.0%
Doubly‐ranked stratification						
First‐order	5.5%	100.0%	100.0%	6.2%	100.0%	100.0%
Modified second‐order	2.2%	100.0%	100.0%	1.6%	100.0%	100.0%
MR‐PRESSO test	2.1%	100.0%	100.0%	1.7%	100.0%	100.0%
Trend test	4.8%	100.0%	100.0%	4.2%	100.0%	100.0%

Results for the residual‐based and doubly‐ranked stratification methods in Table 2 are much better than those for the context‐stratified method, with false positive rates close to 5% for all methods under the null, and power to detect heterogeneity at 100% in almost all cases. The reason for the better performance is that the instrument is stronger in strata from these methods, as the groups are more clearly differentiated according to their exposure distributions. However, when the assumptions for the residual‐based and doubly‐ranked stratification methods are violated (Table 3), they give high false positive rates. In contrast, findings for the context‐stratified method remain largely unchanged.

**TABLE 3:** Simulation study results: proportion of simulated datasets in which either heterogeneity or a trend was detected (defined as the p‐value being less than 0.05) with three different models for the effect of the exposure on the outcome (that is, linear homogeneous effect) and two sets of values of the differences between groups, comparing results from context‐based stratification to previous methods for nonlinear MR when the assumptions for the nonlinear MR methods are violated.

	Larger differences			Smaller differences		
	between subgroups			between subgroups		
	Linear	Quadratic	Threshold	Linear	Quadratic	Threshold
Context‐based stratification						
First‐order	13.5%	60.4%	42.2%	11.5%	29.4%	19.9%
Modified second‐order	0.7%	29.7%	41.7%	0.2%	1.6%	18.6%
MR‐PRESSO test	0.4%	27.7%	41.5%	0.2%	1.5%	17.0%
Trend test	5.2%	60.4%	60.5%	4.7%	33.8%	24.7%
Residual‐based stratification						
First‐order	25.3%	100.0%	100.0%	12.5%	100.0%	100.0%
Modified second‐order	20.1%	100.0%	100.0%	9.1%	100.0%	100.0%
MR‐PRESSO test	20.6%	100.0%	100.0%	9.6%	100.0%	100.0%
Trend test	38.1%	100.0%	97.4%	10.9%	100.0%	100.0%
Doubly‐ranked stratification						
First‐order	94.4%	84.4%	80.7%	91.0%	80.8%	79.6%
Modified second‐order	85.2%	69.8%	80.6%	80.8%	64.7%	79.1%
MR‐PRESSO test	85.7%	71.0%	79.2%	80.9%	64.9%	78.0%
Trend test	99.0%	94.6%	36.5%	98.6%	93.7%	71.9%

**Context‐stratified method in practice:** While using modified second‐order weights in calculation of the Q statistic is necessary to avoid over‐rejection of the null, estimates of the Q statistic with modified second‐order weights for moderate strength instruments are often lower than with first order weights, and hence power to detect true heterogeneity is lower. Users may face a pragmatic choice as to which version of the heterogeneity method to use: whether to use the version with non‐inflated coverage properties under the null, but lower power (modified second‐order or MR‐PRESSO); or the version with better power but inflated coverage under the null (first‐order). The trend test assumes that there is a linear trend between estimates and mean exposure levels in the subgroups, which would not hold if the causal model is U‐shaped. However, if the hypothesized causal model is monotonic in the mean exposure levels (that is, aside from those whose outcome is unchanged by the exposure, the causal effect of the exposure on the outcome is either positive for all individuals in the population, or else it is negative for all individuals in the population), then the trend test will typically have greater power to detect differences in estimates, as was seen in our simulation study. We note that the quadratic function considered in the simulation study is monotonic, as we consider the distribution of the curve away from its turning point.

## 4 Applied Example

We illustrate the use of the context‐stratified MR approach in an applied example. We perform separate MR analyses in recruitment centers from the UK Biobank to investigate the effect of 25‐hydroxyvitamin D [25(OH)D], a circulating metabolite indicating vitamin D levels, on coronary artery disease risk. We selected 25(OH)D as our exposure of interest as its levels depend greatly on environmental context, and on sunlight exposure in particular. The question about a potential effect of vitamin D supplementation, as measured by 25(OH)D, on coronary artery disease risk is a long‐standing one. While randomized trials have suggested no overall effect, it is possible that there is an effect in individuals with low 25(OH)D levels that is masked in the overall analysis.

The UK Biobank is a large prospective cohort of around 500 000 participants aged 40 to 69 years at baseline, recruited across 22 assessment centers throughout the United Kingdom from 2006 to 2010. Our analysis includes data from 20 centers, excluding Stockport (which was the pilot center) and Wrexham (which had much lower participant numbers than other centers). In total, we considered data on 323 539 unrelated European‐ancestry individuals with a valid 25(OH)D measurement using the same exclusion and quality control filters as in a previous article [23]. We use a genetic instrument that is a weighted score comprising 21 variants located in 4 gene regions that have specific biological relevance to vitamin D transport, metabolism, and synthesis, as previously used in the same article. The exposure was shifted to account for the month of blood draw in a similar way to the correction for season of blood draw in the previous article; values represent a measurement taken in October. The outcome was also defined in the same way as in this article, using International Classification of Diseases, Tenth Revision (ICD‐10) codes for fatal ischaemic heart disease (ICD‐10 code: I20‐I25) or nonfatal myocardial infarction (I21‐I23).

Estimates were obtained using the ratio method with a weighted genetic score as the instrument. Genetic associations with 25(OH)D levels were estimated using linear regression. Genetic associations with coronary artery disease were estimated using logistic regression. In all regression models, we adjusted for age at baseline, sex, and 10 genomic principal components of ancestry.

Details of the participants are provided in Table 4. We see that mean 25(OH)D levels vary somewhat across contexts, with lowest levels in the two Scottish recruitment centers, and highest levels in the two Welsh recruitment centers. The difference in mean values from the lowest to the highest center is 7.2 nmol/L, which is around 0.4 standard deviations. In contrast, mean levels of body mass index (BMI), systolic blood pressure (SBP), and low‐density lipoprotein cholesterol (LDL‐c) do not differ as greatly between the centers. This is reassuring for our analysis, as we want to attribute any differences in context‐specific estimates to 25(OH)D levels. However, it means that similar analyses will be difficult for other exposures, particularly for LDL‐c. We assume that groups of the study sample from different centers are exchangeable; that is, they are similar in all ways, and so any difference in estimates is attributable to an effect of vitamin D as measured by 25(OH)D levels.

**TABLE 4:** Baseline characteristics of UK Biobank participants presented as center‐specific means. Centers are ordered based on mean 25‐hydroxyvitamin D [25(OH)D] levels from lowest to highest. For each center, we list the total number of eligible participants (sample size, n), mean age (years), mean 25(OH)D levels (Vit D, nmol/L), mean body mass index (BMI, kg/m^2^), mean systolic blood pressure (SBP, mmHg), and mean low‐density lipoprotein cholesterol (LDL‐c, mmol/L) levels.

Recruitment	Sample size	Age	Vit D	BMI	SBP	LDL‐c
center	(n)	(years)	(nmol/L)	(kg/m^2^)	(mmHg)	(mmol/L)
Glasgow	13 501	56.7	50.2	27.6	139.0	3.49
Edinburgh	12 258	56.7	50.9	27.0	138.5	3.54
Barts	7563	55.6	52.5	26.2	131.0	3.44
Newcastle	23 819	57.2	54.0	27.7	138.5	3.50
Manchester	9502	56.0	54.0	27.4	137.2	3.44
Birmingham	15 779	57.4	54.9	27.7	137.4	3.45
Leeds	29 509	57.1	55.0	27.4	137.8	3.50
Croydon	16 688	57.6	55.2	27.0	134.8	3.47
Stoke	12 843	57.0	55.4	27.7	139.1	3.49
Bury	19 993	57.7	55.5	27.7	137.9	3.45
Oxford	10 499	56.8	55.8	26.6	135.4	3.52
Nottingham	22 831	57.5	56.1	27.4	137.4	3.50
Middlesborough	13 932	57.4	56.1	27.8	141.0	3.53
Liverpool	21 479	57.8	56.3	27.8	137.6	3.44
Hounslow	17 276	57.1	56.4	26.7	134.6	3.43
Sheffield	19 748	57.7	56.8	27.5	138.1	3.50
Reading	21 526	57.0	56.9	26.8	134.9	3.53
Bristol	29 527	56.3	56.9	27.0	138.8	3.52
Swansea	1562	58.1	57.1	28.1	141.6	3.51
Cardiff	12 705	56.4	57.4	27.9	138.5	3.49

**FIGURE 1:**
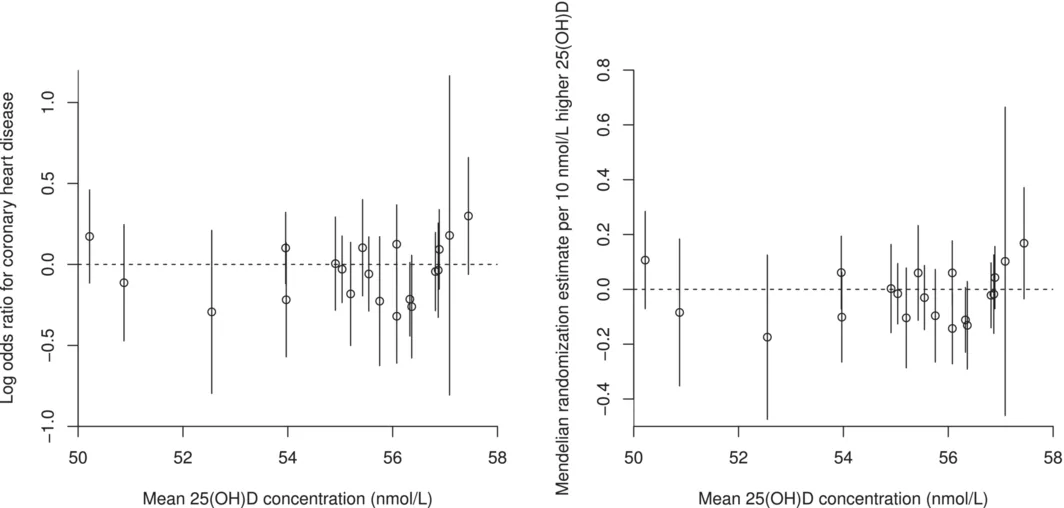
Scatter plots of context‐specific estimates (95% confidence intervals) against mean 25‐hydroxyvitamin D in each subgroup. Left panel: association of weighted genetic score with coronary artery disease risk (log odds ratio). Right panel: MR estimates scaled to 10 nmol/L higher genetically predicted 25(OH)D levels.

Scatter plots of context‐specific estimates against mean exposure levels are provided in Figure 1. In Figure 1 (left panel), we provide the unscaled associations of the weighted genetic score with coronary artery disease risk. In Figure 1 (right panel), we provide the MR estimates scaled to 10 nmol/L higher genetically‐predicted 25(OH)D levels. Estimates in all subgroups are compatible with the null, and there is no evidence of differences in estimates: heterogeneity Q statistic (first‐order) = 22.2, p=0.28; heterogeneity Q statistic (modified second‐order) = 22.2, p=0.28; trend test p=0.76. While there is some variability in the context‐specific estimates, this is no more tha0n would be expected due to chance alone.

**FIGURE 2:**
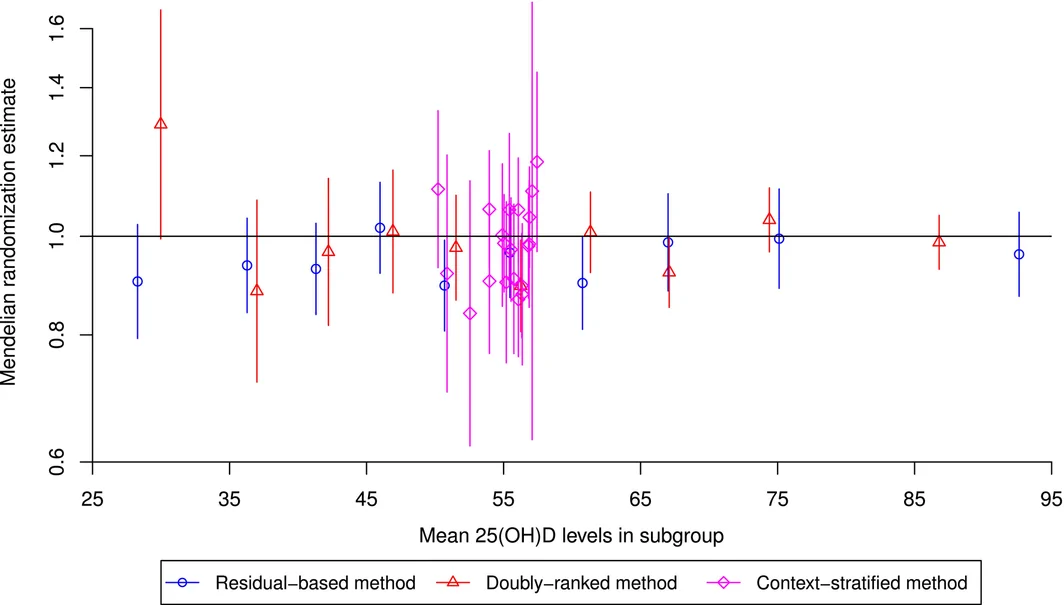
Scatter plots of Mendelian randomization estimates (95% confidence intervals) scaled to 10 nmol/L higher genetically‐predicted 25(OH)D levels against mean 25‐hydroxyvitamin D for subgroups defined by the residual‐based, doubly‐ranked, and context‐stratified methods.

We have previously considered estimates of the effect of 25(OH)D on coronary artery disease risk in 10 subgroups of the population defined using the residual‐based and doubly‐ranked methods [15, 23]. For comparison, we plot our context‐specific estimates on the same axes as these stratum‐specific estimates in Figure 2. We see that estimates from the other stratification methods cover a much wider range than those from the context‐stratified method. While the mean 25(OH)D levels in the strata from the context‐stratified method are all between 50 and 58 nmol/L, mean levels in strata defined by the residual‐based and doubly‐ranked methods range from below 30 to above 85 nmol/L. Considering a wider range of the exposure distribution is desirable, as it provides us with more information on the dose‐response curve.

## 5 Discussion

In this article, we have presented context‐stratified MR as an approach to assess causal effect heterogeneity using MR, and illustrated its use through simulations and an applied example. This approach is conceptually straightforward yet potentially powerful for detecting variability in causal effects across subpopulations defined using an exogenous variable, such as recruitment center. A simulation study showed that the approach can reliably detect nonlinearity, particularly using a trend test in meta‐regression. We illustrated the approach using data from the UK Biobank. While we were not able to detect evidence for a causal effect, let alone heterogeneity in causal estimates, this applied example illustrates the feasibility of the approach for exploring differences in estimates.

### 5.1 Comparison With Previous Methods

Several methods have been proposed for investigating effect heterogeneity and nonlinearity using IVs [24]. Approaches using nonlinear least squares are attractive in some settings [25], but in MR where the IVs typically only explain a small proportion of variance in the exposure, fitted values of the exposure from the first‐stage regression only span a small section of the exposure distribution, meaning that findings from such methods are at best limited in scope. Such approaches can also be sensitive to the specification of the nonlinear model [26]. While flexible nonparametric alternatives have been developed [27, 28, 29], any estimates beyond the exposure values predicted by the IVs rely on blind extrapolation. More promising are approaches that use the residuals from the first‐stage model [30, 31], although again these approaches can be sensitive to parametric assumptions and model specification choices.

Alternatively, several stratification approaches have been proposed, which are more similar to the context‐stratified MR approach discussed here. The residual‐based stratification method calculates residuals from regression of the exposure on the IVs, which represent the value that the exposure would take at a fixed level of the IVs [8, 13]. Stratification on these residual values allows estimates to be calculated for subgroups with large differences in their mean exposure levels. However, the residual‐based stratification method makes a strong and unrealistic assumption (known as the constant‐effect assumption) that the effect of the genetic variants is constant in the population [32]. The doubly‐ranked stratification method is more flexible, stratifying individuals based on their percentile of the counterfactual exposure distribution at a given value of the IVs, under the assumption (known as the rank‐preserving assumption) that this percentile would be the same at all values of the IVs [14]. This is a strictly weaker assumption than the constant‐effect assumption, and is more realistic. However, subgroups defined by the doubly‐ranked method are slightly more similar as to their average levels of the exposure compared to those from the residual‐based method. Additionally, violation of the rank‐preserving assumption would lead to bias [15, 16].

The context‐stratified MR approach makes a more justifiable assumption, relying on the existence of subgroups defined by a natural division of the study population. However, in the example of vitamin D considered in this article, subgroups defined by the context‐stratified approach are far more similar as to their average levels of the exposure than those from other stratification methods. We chose to investigate vitamin D as an exposure as this has plausible variation in levels between centers. However, even for this example, variability in context‐specific mean exposure levels was limited. For other exposures, it may not be possible to find a natural division into subgroups to facilitate this approach. In practice, power to detect heterogeneity in estimates will depend on many factors, including sample size, number of contexts, differences in the exposure level distribution between the contexts, strength of the causal effects, and strength of effects of the instrument on the exposure.

A similar context‐stratified approach was previously performed to investigate the effect of alcohol consumption in different geographic regions of China [33]. However, rather than performing separate analyses in each region, analysts defined a composite instrument score based on genetic variants and geographic region. While they did adjust for geographic region in primary analyses, defining an IV using geographic region could conflate genetic differences and regional differences, such that comparisons are not solely driven by differences in genetic variants.

Other related approaches in MR have considered the comparison of estimates in different subgroups, but typically the focus of these approaches has been to detect or avoid pleiotropic bias rather than to explore effect heterogeneity or nonlinearity. Subgroups in whom the exposure is absent (such as women in analyses investigating the effect of alcohol consumption in East Asia, where women do not tend to drink alcohol for cultural reasons [34]) can serve as natural negative control populations [35]. Gene‐by‐environment interactions can be exploited to account for pleiotropy under certain assumptions about the consistency of pleiotropic effects across subgroups [36]. Additionally, the MR‐GENIUS (MR G‐estimation under No Interaction with Unmeasured Selection) method leverages variance differences in the exposure distribution across genotype subgroups (implicitly due to some unknown interactions) to obtain a single robust estimate [37]. In other work, associations of the lead variant in the *FTO* gene region with BMI have been shown to vary with age [38], and some differences in stratified MR estimates defined according to age and urate levels have been observed [7].

### 5.2 Interpretation of Results

In a randomized controlled trial, differences in subgroup analysis estimates are not necessarily attributable (or not fully attributable) to the stratification trait, as this is not a randomized comparison. For instance, estimates may be higher in men than in women due to greater mean levels of BMI in men, rather than due to any aspect of sex or gender. Similarly in stratified nonlinear MR using the residual‐based or doubly‐ranked method: differences between estimates are not necessarily fully attributable to the exposure, but may be attributable to differences in the strata populations [39]. A similar statement can be made about context‐stratified MR, except that in this approach, subgroups are primarily defined by the context variable. Hence we may expect these differences to be even more marked in context‐stratified MR, as the context strata must by definition differ according to the context variable, and hence potentially differ according to related environmental, social, and cultural factors. Equally, differences in the exposure distribution between subgroups may be less sharp. In the applied example in this article, there is an intrinsic correlation between average exposure levels and geographical location, and hence any differences in estimates may be due to factors that also vary by geographical location. We would urge caution in the interpretation of findings where differences between estimates are detected. We would also encourage analysts to initially describe differences between estimates in neutral terms (for instance, in terms of heterogeneity), and to make clear that any subsequent claim (for instance, nonlinearity or effect modification) is a subjective interpretation for the difference in estimates.

### 5.3 Limitations

Compared with other stratification approaches for nonlinear MR, context‐stratified MR is relatively intuitive. As it does not rely on a technical assumption, but rather exogeneity of the context variable, context‐specific estimates are more likely to be valid causal estimates than stratum‐specific estimates from the residual‐based or doubly‐ranked method, in which inferences depend on more questionable statistical assumptions. However, finding a relevant context variable is likely to be challenging for exposures that do not differ systematically in their distribution across the population. Even for vitamin D, differences in mean levels between UK Biobank centers were not substantial. Care should be taken in context‐stratified MR if the contexts are very small, as genetic variants will be naturally weaker instruments in terms of asymptotic bias with smaller sample sizes. If there is a large number of small contexts, these may be better analyzed in moderate‐sized groups rather than individually. Additionally, differences between estimates in context‐stratified MR are more likely to be explained by another variable other than the exposure compared to estimates from other stratification approaches. Further, as with all methods for making causal inferences, conclusions depend on assumptions that are untestable. When we have a feasible context variable, the assumptions for context‐stratified MR methods are more reasonable than those for other nonlinear MR methods for any given exposure. However, all approaches depend on the validity of the genetic variants as instrumental variables.

### 5.4 Role of Generative Artificial Intelligence in Statistical Research

Given that this manuscript was conceptualized with the aid of AI, we were asked to provide some thoughts on the use of AI in scientific research.

The increasing use of generative AI in scientific research presents both opportunities and challenges. AI tools may assist researchers with tasks such as literature search, coding, data analysis, and the summarization of findings, potentially increasing efficiency and enabling researchers to explore a wider range of ideas. However, their ability to produce fluent and plausible outputs does not imply that those outputs are correct or scientifically informative. Recent work suggests that current AI systems may preferentially generate ideas that are similar to existing scientific knowledge and may be less likely than human researchers to propose null hypotheses or otherwise challenge prevailing assumptions, highlighting the importance of human judgement in identifying questions that are genuinely novel or potentially misguided [40]. Part of the reason for this is the publication gap in the scientific literature: AI models are trained on published research, and so do not have access to the failed ideas, rejected grants, and abandoned notebooks that are instrumental in the training of any scientist.

More generally, AI‐assisted research may create an illusion of understanding, whereby the ability to generate coherent explanations or analyses is mistaken for genuine understanding of the underlying scientific problem [41]. These concerns are particularly important in statistical and medical research, where apparently reasonable errors in data analysis, interpretation, or causal reasoning may lead to misleading conclusions. AI should therefore be viewed primarily as an aid to, rather than a replacement for, scientific judgement. Appropriate safeguards include independent verification of AI‐generated analyses and claims, transparency about the use of AI, protection of confidential and sensitive data, and retention of clear human responsibility for scientific and clinical decisions [42]. The appropriate role of AI may consequently be not simply to automate existing research processes, but to support researchers while preserving the human capacity to question assumptions, recognize uncertainty, and consider explanations that depart from established ways of thinking.

However, given the pace of advancement in AI capability, this advice may be outdated by the time that you are reading it.

### 5.5 Conclusions

In conclusion, context‐stratified MR provides an accessible and conceptually appealing addition to the epidemiologist's toolbox. By exploiting contextual variability in the distribution of the exposure, it allows us to ask questions about effect heterogeneity and nonlinearity. In examples where a relevant context variable can be found, it is a worthwhile exploratory approach to investigate differences in estimates that may be attributable to heterogeneity or nonlinearity in the effect of an exposure. However, differences between estimates may not be attributable to the exposure. Additionally, even in cases where it is feasible, the range of exposure levels in strata defined by the method may be far narrower than those defined by other stratification methods.

## Funding

SB and BARW are supported by the Wellcome Trust (225790/Z/22/Z) and the United Kingdom Research and Innovation Medical Research Council (MC_UU_00040/01). This work was supported by core funding from the British Heart Foundation (RG/F/23/110103), NIHR Cambridge Biomedical Research Centre (NIHR203312), BHF Chair Award (CH/12/2/29428), and by Health Data Research UK, which is funded by the UK Medical Research Council, Engineering and Physical Sciences Research Council, Economic and Social Research Council, Department of Health and Social Care (England), Chief Scientist Office of the Scottish Government Health and Social Care Directorates, Health and Social Care Research and Development Division (Welsh Government), Public Health Agency (Northern Ireland), British Heart Foundation and the Wellcome Trust. This research has been conducted using the UK Biobank Resource under Application Number 98032.

## Conflicts of Interest

The authors declare no conflicts of interest.

## Supporting information




**Data S1**: Supporting Information.

## Data Availability

The data that support the findings of this study are available from UK Biobank. Restrictions apply to the availability of these data, which were used under license for this study.
