# Supplementary material for: Context‐Stratified Mendelian Randomization: Exploiting Regional Exposure Variation to Explore Causal Effect Heterogeneity and Nonlinearity
Source: Stat Med. 2026 Sep 1;45(20-22):e70727. doi: 10.1002/sim.70727 (PMC13534300; doi:10.1002/sim.70727)
Supplement: Supplementary file 1 [file SIM-45-0-s001.pdf]

Supplementary Material

A.1 Artificial intelligence prompt

The prompt to write the initial draft of this paper was as follows: "Could you please write a draft of a scientific paper that proposes a novel paradigm for Mendelian randomization by exploiting regional variation in the distribution of the risk factor of interest? The idea would be to perform separate Mendelian randomization analyses in different study populations that have different average levels of the exposure, and to use this to explore heterogeneity (and potentially, non-linearity) in effect estimates. The draft should have an Abstract, Introduction, Methods, Applied example, Results, and Discussion sections. The applied example will investigate the effect of vitamin D supplementation on coronary artery disease risk in different UK Biobank recruitment centres. There is no need to perform these analyses, but please leave space for us to include those analyses in the manuscript. Please clarify how this approach differs from other GxE methods, such as MR-Genius. The paper should be formatted for Statistics in Medicine, and aimed at an audience of applied statisticians and quantitative epidemiological researchers. The method could be called "dynamic contextual Mendelian randomization", but if you have a better name then we are happy to consider. Please provide an editable manuscript file, and code (in the R programming language) for running simulations to include in the submission (if you include simulations as part of the manuscript). We will read and edit the draft thoroughly before submission. Thank you for your help."

A.2 Simulation study code

We here report the simulation study code, implemented using the R statistical software language:

```
library(meta)
library(RadialMR)
library(MendelianRandomization)

scens = 3
times = 1000
parts = 10000
groups = 10

set.seed(496)

alpha = rep(seq(from=8, by=0.2, length.out=groups), each = parts)
 # larger differences
alpha = rep(seq(from=9, by=0.1, length.out=groups), each = parts)
 # smaller differences
subgroup = rep(1:10, each = parts)
q1.val = NULL; q1.pval = NULL; qq1.val = NULL;
qq1.pval = NULL; trend1.pval = NULL;
q2.val = NULL; q2.pval = NULL; qq2.val = NULL;
qq2.pval = NULL; trend2.pval = NULL;
q3.val = NULL; q3.pval = NULL; qq3.val = NULL;
qq3.pval = NULL; trend3.pval = NULL;

 for (j in 1:times) {
    bx = NULL; by1 = NULL; by1se = NULL;
  bxse = NULL; by2 = NULL; by2se = NULL;
 xmean = NULL; by3 = NULL; by3se = NULL;
     g = rbinom(parts*groups, 2, 0.3)
     u = rnorm(parts*groups)
  epsx = rnorm(parts*groups)
 epsy1 = rnorm(parts*groups)
 epsy2 = rnorm(parts*groups)
 epsy3 = rnorm(parts*groups)
     x = alpha + 0.5*g + u + epsx
    y1 = 0.8*x - u + epsy1
    y2 = 0.04*x^2 - u + epsy2
    y3 = 0.25*ifelse(x>10, 1, 0)*(x-10) - u + epsy3
 for (k in 1:groups) {
 xmean[k] = mean(x[subgroup==k])
   bx.reg = summary(lm(x[subgroup==k]~g[subgroup==k]))
    bx[k] = bx.reg$coef[2]
  bxse[k] = bx.reg$coef[2,2]
  by1.reg = summary(lm(y1[subgroup==k]~g[subgroup==k]))
   by1[k] = by1.reg$coef[2]
```

```
 by1se[k] = by1.reg$coef[2,2]
  by2.reg = summary(lm(y2[subgroup==k]~g[subgroup==k]))
   by2[k] = by2.reg$coef[2]
 by2se[k] = by2.reg$coef[2,2]
  by3.reg = summary(lm(y3[subgroup==k]~g[subgroup==k]))
   by3[k] = by3.reg$coef[2]
 by3se[k] = by3.reg$coef[2,2]
   }

          est1 = sum(by1*bx*by1se^-2)/sum(bx^2*by1se^-2)
     q1.val[j] = sum( (by1/bx-est1)^2*(by1se/bx)^-2 )
          reg1 = metagen(by1/bx, by1se/bx)
         mreg1 = metareg(reg1, ~xmean)
    q1.pval[j] = reg1$pval.Q
 trend1.pval[j] = mreg1$pval[2]
          rad1 = ivw_radial(mr_input(bx, bxse, by1, by1se), weights=3,
                            alpha=1e-100, summary=FALSE)
    qq1.val[j] = rad1$q
   qq1.pval[j] = pchisq(rad1$q, df=groups-1, lower.tail=FALSE)

   df1 = data.frame(beta.outcome = by1, beta.exposure = bx,
                    se.outcome = by1se,   se.exposure = bxse)

presso1 <- mr_presso(BetaOutcome = "beta.outcome",
                     BetaExposure = "beta.exposure",
                     SdOutcome = "se.outcome",
                     SdExposure = "se.exposure",
                     data = df1,
                     NbDistribution = 1000,
                     SignifThreshold = 0.05)
   qqq1.val[j]  = presso1$`MR-PRESSO results`$`Global Test`$RSSobs
   qqq1.pval[j] = presso1$`MR-PRESSO results`$`Global Test`$Pvalue


   est2 = sum(by2*bx*by2se^-2)/sum(bx^2*by2se^-2)
 q2.val[j] = sum( (by2/bx-est2)^2*(by2se/bx)^-2 )
   reg2 = metagen(by2/bx, by2se/bx)
  mreg2 = metareg(reg2, ~xmean)
 q2.pval[j] = reg2$pval.Q
 trend2.pval[j] = mreg2$pval[2]
          rad2 = ivw_radial(mr_input(bx, bxse, by2, by2se), weights=3,
                            alpha=1e-100, summary=FALSE)
    qq2.val[j] = rad2$q
   qq2.pval[j] = pchisq(rad2$q, df=groups-1, lower.tail=FALSE)
       df2 = data.frame(beta.outcome = by2, beta.exposure = bx,
                    se.outcome = by2se,   se.exposure = bxse)

presso2 <- mr_presso(BetaOutcome = "beta.outcome",
```

```
                     BetaExposure = "beta.exposure",
                     SdOutcome = "se.outcome",
                     SdExposure = "se.exposure",
                     data = df2,
                     NbDistribution = 1000,
                     SignifThreshold = 0.05)
    qqq2.val[j]  = presso2$`MR-PRESSO results`$`Global Test`$RSSobs
    qqq2.pval[j] = presso2$`MR-PRESSO results`$`Global Test`$Pvalue


   est3 = sum(by3*bx*by3se^-2)/sum(bx^2*by3se^-2)
 q3.val[j] = sum( (by3/bx-est3)^2*(by3se/bx)^-2 )
   reg3 = metagen(by3/bx, by3se/bx)
  mreg3 = metareg(reg3, ~xmean)
 q3.pval[j] = reg3$pval.Q
 trend3.pval[j] = mreg3$pval[2]
          rad3 = ivw_radial(mr_input(bx, bxse, by3, by3se), weights=3,
                            alpha=1e-100, summary=FALSE)
     qq3.val[j] = rad3$q
    qq3.pval[j] = pchisq(rad3$q, df=groups-1, lower.tail=FALSE)

        df3 = data.frame(beta.outcome = by3, beta.exposure = bx,
                    se.outcome = by3se,   se.exposure = bxse)

presso3 <- mr_presso(BetaOutcome = "beta.outcome",
                     BetaExposure = "beta.exposure",
                     SdOutcome = "se.outcome",
                     SdExposure = "se.exposure",
                     data = df3,
                     NbDistribution = 1000,
                     SignifThreshold = 0.05)
    qqq3.val[j]  = presso3$`MR-PRESSO results`$`Global Test`$RSSobs
    qqq3.pval[j] = presso3$`MR-PRESSO results`$`Global Test`$Pvalue

   }

sum(q1.pval<0.05)
sum(q2.pval<0.05)
sum(q3.pval<0.05)
sum(qq1.pval<0.05)
sum(qq2.pval<0.05)
sum(qq3.pval<0.05)
sum(qqq1.pval<0.05)
sum(qqq2.pval<0.05)
sum(qqq3.pval<0.05)
sum(trend1.pval<0.05)
sum(trend2.pval<0.05)
sum(trend3.pval<0.05)
```
